# Hepatic Expression of Growth Hormone Receptor (*GHrec*) and Insulin-like Growth Factor-I (*IGF-I*) Genes and Cellular Location of *IGF-I* mRNA in Diploid and Triploid Atlantic Salmon (*Salmo salar*) Undergoing Parr–Smolt Transformation

**DOI:** 10.3390/ani16030515

**Published:** 2026-02-06

**Authors:** Martina Bortoletti, Elisa Fonsatti, Lisa Maccatrozzo, Stefano Peruzzi, Malcolm Jobling, Marta Vascellari, Giuseppe Radaelli, Daniela Bertotto

**Affiliations:** 1 Department of Comparative Biomedicine and Food Science, University of Padova, 35020 Legnaro, Italy; martina.bortoletti@unipd.it (M.B.); elisa.fonsatti@unipd.it (E.F.); lisa.maccatrozzo@unipd.it (L.M.); daniela.bertotto@unipd.it (D.B.); 2 Department of Arctic and Marine Biology, UiT the Arctic University of Norway, 9019 Tromsø, Norway; stefano.peruzzi@uit.no (S.P.); malcolm.jobling@uit.no (M.J.); 3 Department of Histopathology, Istituto Zooprofilattico Sperimentale delle Venezie, 35020 Legnaro, Italy; mvascellari@izsvenezie.it

**Keywords:** *Salmo salar*, parr–smolt transformation, polyploidy, gene expression, insulin-like growth factor I (*IGF-I*)

## Abstract

The induction of triploidy, a strategy to mitigate unwanted pre-harvest sexual maturation and a genetic containment measure for escaped farmed Atlantic salmon (*Salmo salar*), may give rise to challenges because of the distinct environmental and dietary requirements of sterile triploid fish. Smoltification is a critical phase in the life cycle of Atlantic salmon, so knowledge about parr–smolt transformation in triploids is important for the salmon farming industry. This study covered an investigation of hepatic expression of growth hormone receptor (*GHrec*) and insulin-like growth factor-I (*IGF-I*) genes, both of which are intimately involved in the regulation of osmoregulation and growth. Additionally, hepatic presence and location of *IGF-I* mRNA were examined using RNAscope^®^, an advanced in situ hybridization technique. Triplicate groups of juvenile diploid and triploid salmon were reared at low temperature (10 °C) and fed either a standard diet or one enriched with hydrolyzed fish proteins from the start of feeding onwards. Liver samples were collected from three fish per tank each month from October to December (2454–3044 degree-days post-start feeding), the period encompassing smoltification, and hepatic expression of *IGF-I* and *GHrec* genes was quantified by real-time PCR. The results indicated that neither ploidy nor diet significantly influenced *IGF-I* or *GHrec* gene expression, suggesting that, under our conditions, triploidy and diet did not adversely affect this molecular pathway linked to growth and osmoregulation. *IGF-I* gene expression exhibited significant temporal variation, correlating with the progression of smoltification, while *GHrec* gene expression showed a similar, albeit non-significant, trend. Triploids exhibited *IGF-I* and *GHrec* gene expression patterns comparable to diploids, and both the temporal changes and lack of difference between triploids and diploids were mirrored in the quantification of *IGF-I* mRNA within the liver cells. The potential applicability to a commercial aquaculture setting requires further investigation.

## Simple Summary

Farmed Atlantic salmon are made sterile to prevent them from breeding with wild fish if they escape. These sterile fish, called triploids, may have different needs than normal salmon. One of the most delicate stages in a salmon’s life is the transition from freshwater to seawater, known as smoltification. If this process is not well timed, fish may struggle with growth, health, and survival after they are moved to the sea. In this study, we examined whether triploids and normal salmon differ in how their livers respond during this transition. We also tested whether a diet containing hydrolyzed fish proteins influences this response. We measured two genes in the liver that help control growth and help the fish adapt to salty water, and we also looked at where signals from one of these genes appear inside liver cells. We found no major differences between triploids and normal salmon, and the diet had no effect. Instead, both groups showed clear natural changes as smoltification progressed. These findings suggest that triploid salmon cope with this life stage in the same way as normal fish and that these genes could help identify the best moment to move fish to the sea.

## 1. Introduction

Atlantic salmon (*Salmo salar*) farming is a significant part of the global aquaculture industry, with production reaching approximately 2.87 million metric tons in 2023 [1]. Norway maintains its position as the world’s leading producer, contributing substantially to the European aquaculture sector, which generates over €2.5 billion in value from Atlantic salmon alone [2]. Nevertheless, the Atlantic salmon farming industry continues to face significant challenges, including high mortality that sometimes occurs shortly after the transfer of the fish from freshwater to the sea [3,4].

The parr–smolt transformation, also known as smoltification, is a critical process in the life cycle of Atlantic salmon, significantly impacting performance, welfare, and survival. It involves morphological, physiological, and behavioral changes that enable young salmon (parr) to transform into smolts that can survive and grow in the marine environment [5]. Smolts develop silver skin pigmentation and a streamlined body [5,6], and they are characterized by increased metabolic rate, enhanced seawater tolerance, and alterations in lipid metabolism compared to parr [7]. Behavioral changes include downstream movement and the loss of territorial behavior [6,8,9].

The parr–smolt transformation is orchestrated by an interplay of hormones, primarily growth hormone (GH), cortisol, and insulin-like growth factor I (*IGF-I*) [5,10], and it is influenced by environmental cues such as photoperiod and temperature [11,12,13]. The physiological effects of GH are mediated through its transmembrane receptor (*GHrec*), which activates intracellular signaling pathways regulating growth, metabolism, and osmoregulatory processes in target tissues, with the liver representing a primary site of action [14,15]. Environmental cues trigger hypothalamus-mediated GH secretion from the pituitary gland, which stimulates *IGF-I* synthesis and secretion. *IGF-I* production is highest in the liver and may be elevated after seawater adaptation [16]. *IGF-I* acts as a key mediator of GH action by promoting growth and metabolic regulation and plays an important role during smoltification in coordination with other endocrine factors [17,18]. GH and cortisol initiate long-term adaptations to the new osmotic environment, while *IGF-I* promotes the hyperplasia and hypertrophy of transporting cells and vasculature in osmoregulatory tissues [5]. Based on their central role in the GH/IGF endocrine axis and their involvement in growth regulation and osmoregulatory adaptation during smoltification, *IGF-I* and *GHrec* were selected in this study as molecular markers to assess physiological responses in diploid and triploid Atlantic salmon.

The induction of triploidy may add another layer of complexity to the parr–smolt transformation. Farming of sterile triploid Atlantic salmon mitigates ecological concerns associated with escaped farmed fish and prevents the adverse effects of pre-harvest sexual maturation [19,20]. However, the altered physiology of triploids can result in differences in environmental adaptability and nutritional needs, affecting their growth and welfare compared to diploids, particularly after seawater transfer [21,22]. Furthermore, triploidy may influence parr–smolt transformation [23].

In a previous study [24], biomarkers related to growth, osmoregulation, and stress followed similar patterns in young triploid and diploid salmon. Both diploids and triploids appeared to successfully complete the parr–smolt transformation, as evidenced by decreased condition factor, reduced plasma chloride concentration and osmolality following seawater challenge tests, and temporal changes in muscular gene expression of *IGF-I*, GH, and growth hormone receptor (*GHrec*).

The present study builds upon these findings, aiming to further evaluate the effects of triploidy and diet on Atlantic salmon parr–smolt transformation, with a focus on the liver. The liver plays a vital role in metabolic processes, including the production and regulation of hormones involved in growth and osmoregulation [25,26]. Moreover, it exhibits the highest levels of *IGF-I* and *GHrec* gene expression in fish [14,27,28,29,30]. Therefore, assessing the hepatic expression of these genes during the parr–smolt transformation may provide insights into this developmental process.

To enhance our investigation, we employed RNAscope^®^, a sensitive multiplex nucleic acid technology that allows detection and visualization of mRNA in formalin-fixed paraffin-embedded organs [31,32,33,34]. This enabled us to study the presence and location of *IGF-I* mRNA in the liver cells.

Our study aims to test whether triploidy and diet differentially affect the hepatic expression of *IGF-I* and *GHrec* genes during parr–smolt transformation in Atlantic salmon. Specifically, our objectives were to determine if there are significant differences in the hepatic expression levels of *IGF-I* and *GHrec* genes between diploid and triploid Atlantic salmon during parr–smolt transformation, assess whether different dietary compositions result in different expression patterns, investigate the cellular location of *IGF-I* mRNA within liver cells, and quantify the mRNA signals.

## 2. Materials and Methods

### 2.1. Experimental Design, Fish Rearing, and Sample Collection

The experimental design, fish production, diet formulation, and rearing conditions were those described in Peruzzi et al. (2018) [35]. Briefly, diploid and triploid Atlantic salmon (*Salmo salar*) were reared in triplicate 200 L circular indoor tanks (initially ~3000 fish per tank, ~620 g biomass) and fed either a standard fish meal (STD) diet or a modified diet with 45% of fish meal replaced by hydrolyzed fish proteins (HFM) (Skretting AS, Stavanger, Norway) from the time of start-feeding, resulting in four experimental groups: diploid and triploid fish fed either STD or HFM diets.

Fish were maintained at low temperature (10 ± 0.5 °C) from start-feeding through parr–smolt transformation when they were transferred to 500 L tanks. Constant light conditions were employed throughout the experiment, except for a period of reduced day length (08L:16D) from September to October to simulate winter conditions and induce parr–smolt transformation [35].

Samplings were conducted monthly from October to December (2454–3044 degree-days post-start feeding, ddPSF), encompassing the parr–smolt transformation period. At each sampling time, liver samples (1 cm × 1 cm) were collected from 9 fish from each treatment group (3 fish per tank and 3 tanks for each treatment; body weights of fish are reported in the Supplementary Materials, Table S1). Samples were quickly frozen on dry ice and stored at −80 °C for subsequent gene expression analysis. Additional samples (*n* = 20) were fixed in paraformaldehyde (0.1 M phosphate-buffered saline, pH 7.4), dehydrated, and embedded in paraffin for in situ hybridization. Serial sections (4 μm thick) were obtained using a microtome and processed with RNAscope^®^ to investigate the presence and cellular location of *IGF-I* mRNA.

### 2.2. Gene Expression Analysis

#### 2.2.1. RNA Extraction and cDNA Synthesis

Total RNA was extracted from liver samples collected at three time points: October (2454 ddPSF), November (2745 ddPSF), and December (3044 ddPSF), using TRIZOL reagent (Life Technologies, Carlsbad, CA, USA) according to the manufacturer’s instructions. RNA concentration was quantified using a NanoDrop 2000 spectrophotometer (Thermo Fisher Scientific™, Waltham, MA, USA).

Prior to cDNA synthesis, samples were treated with DNase using a DNase I, Amplification Grade Kit (Invitrogen™, Waltham, MA, USA). First-strand cDNA was then synthesized from 1 μg of total RNA using SuperScript II Reverse Transcriptase (Invitrogen™, Carlsbad, CA, USA) and random hexamer primers (10 μM) (Thermo Fisher Scientific™, Waltham, MA, USA) in a total reaction volume of 22 μL. All protocols were performed according to the manufacturer’s instructions.

#### 2.2.2. Real-Time Quantitative PCR

Specific primer sequences for the target genes (*IGF-I* and *GHrec*) and for the reference gene β-actin (*β-ACT*) were designed using Primer Express software version 3.0 (Applied Biosystems, Life Technologies, Carlsbad, CA, USA) and are presented in Table 1. The PCR reaction mixture (25 μL total volume) consisted of 12.5 μL of PowerSYBR^®^ Green PCR Master Mix (Applied Biosystems by Thermo Fisher Scientific™, Waltham, MA, USA), 0.75 μL each of forward and reverse primers (10 μM), 8.5 μL RNAase-free water, and 2.5 μL cDNA. All reactions were performed in triplicate, including negative controls, using a real-time PCR 7500 thermal cycler (Applied Biosystems, Life Technologies, Carlsbad, CA, USA) and analyzed with 7500 Software v2.0.5 (Applied Biosystems, Life Technologies, Carlsbad, CA, USA).

The PCR amplification protocol was as follows: (1) 50 °C for 2 min, (2) 95 °C for 10 min, (3) 95 °C for 15 s, and (4) 60 °C for 1 min. Steps 3–4 were repeated for 39 additional cycles, for a total of 40 cycles.

Following the amplification, a dissociation stage was included for melting curve analysis, which was performed over a range of 60–95 °C (increment of 0.5 °C for 2 s) to detect nonspecific products and/or primer dimers. The mRNA expression levels were normalized against *β-ACT* expression, which served as the housekeeping gene. Quantitative gene expression levels were calculated using the 2^−ΔΔCt^ method [36].

### 2.3. RNAscope^®^ Analysis

#### 2.3.1. RNAscope^®^ In Situ Hybridization for *IGF-I* mRNA Detection

RNAscope^®^ in situ hybridization (ISH) was performed on a subset of liver sections from ten fish sampled in both October and November, previously identified as the critical months for Atlantic salmon parr–smolt transformation [24]. The procedure was carried out using a Ventana Discovery Ultra autostainer (Ventana Medical System, Roche, Tucson, AZ, USA), following the manufacturer’s protocol.

The RNAscope^®^ 2.5 VS specific Probe Ssa-LOC100136517 (Advanced Cell Diagnostics Inc., Santa Monica, CA, USA; Cat. No. 902109) was employed, targeting the region 2–712 of *Salmo salar* (Ssa) *IGF-I* mRNA (Accession No: NM_001123623.1). Formalin-fixed, paraffin-embedded (FFPE) serial sections (4 µm thick) were deparaffinized and pre-treated with heat (97 °C for 24 min) and protease (ACD Kit RNAscope VS Universal AP cod. 323250, Advanced Cell Diagnostics Newark, CA, USA) prior to hybridization. The probe was incubated at 42 °C for 2 h.

Hybridization signals were visualized as red, punctate precipitates using the mRNA RED Detection Kit (Roche cod. 07099037001). For each sample, two additional sections were processed: one using probes for Ssa-PPIB [peptidylprolyl isomerase B (cyclophilin B)] as an endogenous control to assess RNA integrity, and another using dapB [*Bacillus subtilis* dihydrodipicolinate reductase gene] as a negative control to evaluate background staining [31,37].

#### 2.3.2. Quantification of RNAscope^®^ ISH Signals for *IGF-I* mRNA

**Figure 1:**
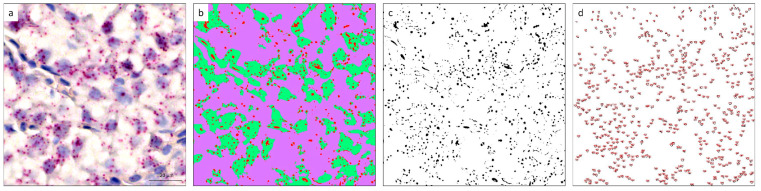
Image processing workflow for quantification of *IGF-I* mRNA in salmon liver using RNAscope^®^ and ImageJ (Fiji) analyses. (**a**) Representative liver section image (40× magnification). (**b**) Segmented image following Trainable Weka Segmentation. (**c**) Thresholded image highlighting *IGF-I* mRNA signals. (**d**) Quantification of discrete *IGF-I* mRNA signals (red dots) using the analyze particles function. Scale bar: 20 µm.

Slide images were captured using an Aperio LV1 IVD slide scanner (Leica Biosystems, Milano, Italy) at 40× magnification (Figure 1a). Quantitative analysis of mRNA signals in October and November samples was performed using ImageJ software (version 2.7.0 Fiji) [38].

The “Trainable Weka Segmentation” plugin was employed to classify areas based on three color categories (Figure 1b): (i) *IGF-I* mRNA signals (red), (ii) nuclei stained with hematoxylin (green), and (iii) background (purple). A reference sample image was used for this classification. The *IGF-I* color class was then isolated using the “threshold” function (Figure 1c). Subsequently, images were subjected to a “watershed” function to separate signal clusters, and the “Analyse particles” function was used to estimate the number of positive signals per image (Figure 1d).

Quantification was performed on ten 100 × 100 µm^2^ tissue areas per slide, with each slide representing an individual animal (*n* = 10).

### 2.4. Statistical Analysis

All data were analyzed using R software (version R 4.3.2) [39]. Data were assessed for normality, and outliers were removed prior to analysis. Initial analysis of the gene expression data used a linear mixed model, which included sampling time, ploidy, and diet as fixed factors and tank as a random factor. In cases where the analyses revealed a non-significant effect, factors were removed from the statistical model. The data were then re-analyzed using a two-way ANOVA with sampling time and ploidy as main factors, followed by Tukey’s post hoc test.

RNAscope^®^ ISH data were analyzed using a mixed-effects model with sampling time as a fixed effect and slide (corresponding to individual animals) as a random factor. Results are presented as mean ± standard error (SE). Differences among means were considered statistically significant at *p* < 0.05.

## 3. Results

### 3.1. Gene Expression Analysis

**Figure 2:**
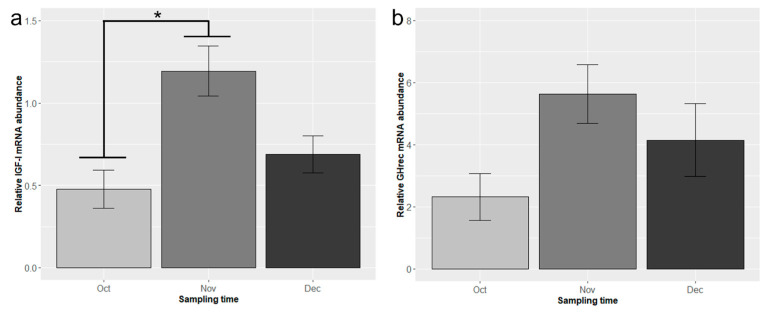
Hepatic mRNA expression of (**a**) insulin-like growth factor I (*IGF-I*) and (**b**) growth hormone receptor (*GHrec*) in diploid and triploid Atlantic salmon during the parr–smolt transformation. Data are presented as mean ± standard error (SE). Statistically significant differences are indicated by an asterisk * (*p* < 0.05).

The statistical analysis revealed that diet and tank had no significant effect on gene expression. Consequently, these factors were removed from the statistical model, and the data were re-analyzed. The hepatic expression of insulin-like growth factor I (*IGF-I*) was significantly influenced by sampling time, but not by ploidy (Figure 2a). *IGF-I* mRNA levels increased significantly from October to November (*p* < 0.05), followed by a non-significant decrease from November to December.

Growth hormone receptor (*GHrec*) expression showed a similar trend to *IGF-I*, with an increase from October to November and a slight decrease from November to December. This increase was particularly pronounced in diploid fish, approaching statistical significance (*p* = 0.07). However, overall changes were not statistically significant, and there was no effect of ploidy (Figure 2b).

### 3.2. Cellular Localization and Quantification of IGF-I mRNA Using RNAscope^®^ Technology

**Figure 3:**
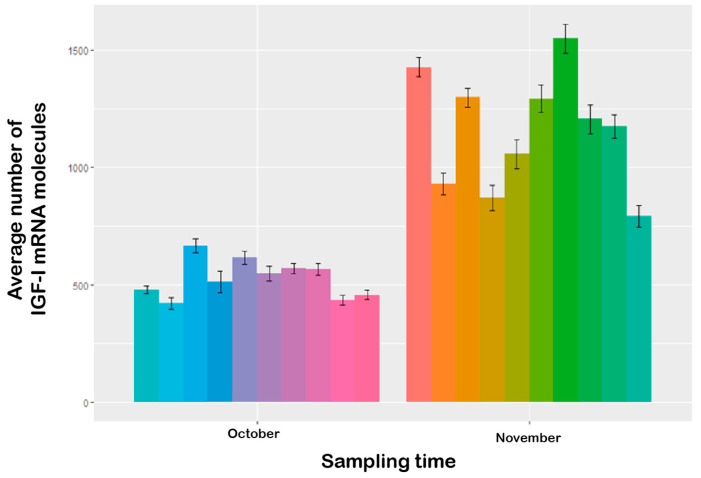
Quantitative analysis of *IGF-I* mRNA using RNAscope^®^ in situ hybridization in Atlantic salmon liver. Mean number of *IGF-I* mRNA signals detected per animal (*n* = 10) in October and November, with 10 counts per animal. Each bar represents a different animal, distinguished by unique colors. Error bars represent standard error (SE).

**Figure 4:**
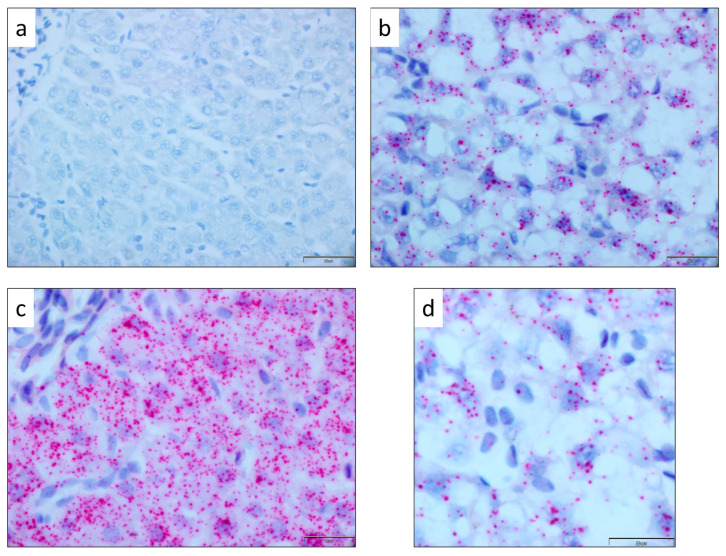
Qualitative analysis of *IGF-I* mRNA using RNAscope^®^ in situ hybridization in Atlantic salmon liver. Representative images of RNAscope^®^ in situ hybridization in liver parenchyma: (**a**) negative control; (**b**) sample from October, with (**d**) detail showing a higher-contrast view of *IGF-I* mRNA signal distribution; and (**c**) sample from November. Red spots indicate discrete *IGF-I* mRNA signals. Nuclei are counterstained with hematoxylin. Scale bar: 20 µm. Images taken at 40× magnification.

Quantitative analysis revealed a significantly higher mean number of *IGF-I* mRNA signals in November (1159.51 ± 28.30) than in October (527.34 ± 11.27; *p* < 0.0001; Figure 3). All samples showed good mRNA integrity when incubated with the PPIB probe and were negative for the DapB control probe (Figure 4a), confirming the specificity and reliability of the assay. The *IGF-I* mRNA signal was visualized as multiple small red dots with nuclear and perinuclear localization (Figure 4d), diffusely distributed among hepatocytes. The number of dots per cell varied between sampling times, with November samples (Figure 4c) exhibiting a higher signal density compared to October samples (Figure 4b), consistent with the quantitative analysis.

## 4. Discussion

This study, investigating the hepatic expression of genes involved in fish growth and osmoregulation during Atlantic salmon parr–smolt transformation, complements and extends the published work on growth [35], liver transcriptome and histology [40], digestive tract morphology and enzyme activities [41], and physiological indicators and biomarkers [24] carried out on the same treatment groups of fish. The focus was on the hepatic expression of insulin-like growth factor I (*IGF-I*) and growth hormone receptor (*GHrec*), using real-time PCR and RNAscope^®^ in situ hybridization. Our findings point to the liver being a relevant tissue for investigation and playing a role in the regulation of physiological processes that occur during the parr–smolt transformation [42].

### 4.1. Effects of Ploidy and Diet

Neither ploidy nor diet significantly affected gene expression during parr–smolt transformation. These findings indicate the efficacy of low-temperature rearing conditions and phosphorus-rich diets in mitigating any differences between diploids and triploids. These results align with previous studies demonstrating that the physiological requirements of triploids can be met by adjusting water temperature and diet composition [23,24,25,43,44,45,46].

### 4.2. Temporal Changes in Gene Expression

While ploidy and diet effects were negligible, our study revealed significant temporal changes in hepatic gene expression during the parr–smolt transformation (Figure 2), which increased from October (2454 ddPSF) to November (2745 ddPSF), followed by a non-significant decrease from November to December (3044 ddPSF) (Figure 2). This is consistent with the significant up-regulation of hepatic genes correlated with metabolic and growth processes previously observed in diploid and triploid parr vs. smolts in some of the same treatment groups of fish (STD diet) [40].

The observed temporal pattern (Figure 2) is mirrored in physiological changes during smolt development and corresponds with the findings of our previous study on muscular gene expression [24]. In both studies, we observed that *IGF-I* and *GHrec* expression peaked during parr–smolt transformation in November, with a consistent pattern across tissues (liver and muscle).

The endocrine control of the parr–smolt transformation involves multiple hormones, including GH and *IGF-I*, and their receptors, which stimulate seawater adaptation and osmoregulatory changes [47]. Plasma concentrations of these hormones temporarily increase [48], stimulating chloride cell proliferation and/or enlargement [49] and enhancing gill ion transporter enzyme activity [12]. The findings of our study on hepatic expression of *GHrec* and *IGF-I* are in line with these established endocrine patterns. Our observations of sustained hepatic *GHrec* expression, even as fish approached full seawater adaptation, corroborate previous findings that GH receptor density in the liver and other organs remains elevated after plasma GH levels return to freshwater levels [5]. GH receptors are present in various osmoregulatory organs besides the liver, suggesting that GH may act on these organs either directly or via local *IGF-I* release [49]. Consistently, *IGF-I* mRNA has been detected in various osmoregulatory organs, with enhanced expression observed after seawater transfer of several species of salmonids [49].

### 4.3. Molecular Markers of Parr–Smolt Transformation

The distinct hepatic expression patterns of *IGF-I* and *GHrec* observed in our study (Figure 2) suggest that these could serve as molecular biomarkers of parr–smolt transformation in Atlantic salmon. The findings align with those of Shwe et al. [40], who identified differentially expressed genes and enriched biological pathways in the liver of Atlantic salmon during parr–smolt transformation. Together, the findings suggest that changes in hepatic gene expression could serve as molecular indicators for assessing smolt readiness for transfer to seawater, in conjunction with established methods such as seawater challenge tests, gill Na^+^/K^+^-ATPase (NKA) mRNA expression and enzymatic activity measurements, and plasma concentrations of smolt-related hormones [10,50,51]. Importantly, our findings of similarity in gene expression patterns between diploid and triploid salmon indicate that these molecular markers could be equally applicable to both ploidy levels.

The peak expression of the hepatic genes during November (2745 ddPSF) (Figure 2) suggests this may have coincided with the optimal time for transfer of the fish to seawater. This aligns with the work of Harvey et al. [52], who characterized genome regulation in salmon liver during the parr–smolt transformation. Their integrated analyses revealed a marked upregulation of genes involved in ribosome biogenesis and protein synthesis pathways in the fish just prior to seawater entry. The authors linked this increased protein synthesis capacity to anticipated higher circulating growth hormone levels after seawater migration. Since GH and *IGF-I* are part of the same hormonal axis regulating growth and protein anabolism, the peak expression observed in our November samples is consistent with the preparatory upregulation of the translation machinery reported by Harvey et al. [52].

### 4.4. RNAscope^®^ Analysis

RNAscope^®^ analysis allowed visualization of *IGF-I* mRNA as discrete signals with nuclear and perinuclear localization in hepatocytes, providing spatial context to the expression patterns. The hepatic parenchyma of November samples exhibited markedly higher positivity to the *IGF-I* probe compared to October samples (Figure 3), mirroring the seasonal trend observed in the gene expression analysis (Figure 2). Quantitative estimation of *IGF-I* mRNA abundance revealed a significantly higher number of *IGF-I* mRNA signals in November compared to October (Figure 3), corroborating the PCR results. This substantial increase underscores the considerable upregulation of *IGF-I* expression during the parr–smolt transformation.

These findings align with the known actions of *IGF-I*, GH, and cortisol, acting together to improve salinity tolerance and playing crucial roles in osmoregulation and seawater adaptation [48]. The increased *IGF-I* expression observed in November (2745 ddPSF), both at the transcriptional level and in terms of spatial distribution within hepatocytes, underscores its importance in preparing Atlantic salmon for the physiological challenges associated with seawater entry.

The consistency between our PCR and RNAscope^®^ results provides robust evidence that *IGF-I* plays a role in the regulation of the physiological processes involved in parr–smolt transformation of Atlantic salmon. The depth of information provided by RNAscope^®^ makes it a valuable tool for research purposes. Future studies could utilize this technique to investigate tissue distributions of the molecular components known to be involved in parr–smolt transformation, potentially leading to more refined molecular markers for assessing smolt readiness for transfer from freshwater to seawater.

## 5. Conclusions

In summary, our analysis of hepatic expression of key genes involved in fish growth and osmoregulation, complemented by RNAscope^®^ visualization, revealed significant temporal changes during Atlantic salmon parr–smolt transformation. Peak gene expression was observed in November (2745 ddPSF), a pattern consistent across ploidies and unaffected by diet type (standard or hydrolyzed fish protein). These findings suggest that the rearing conditions successfully minimized ploidy-related molecular differences. The liver’s role in parr–smolt transformation was demonstrated by our findings, indicating its value as an organ for studying biomarkers linked to parr–smolt transformation. The observed temporal changes in *IGF-I* and *GHrec* expression align with the physiological preparations necessary for seawater adaptation, and additional supportive evidence was provided by quantification of *IGF-I* mRNA in the liver cells. Future research could focus on validating these markers across different environmental conditions and exploring their applicability in commercial aquaculture settings.

## Figures and Tables

**Table 1:** Primer sequences used.

Gene	Forward Primer (5′–3′)	Reverse Primer (5′–3′)
*IGF-I*	5′-CGGTCACATAACCGTGGTATTG-3′	5′-CTGCCTTGCCAGACTTGACA-3′
*GHrec*	5′-GGAAGACATCGTGGAACCAGA-3′	5′-CAAACTGGCTCCCGGTTAGA-3′
*β-ACT*	5′-CAGCCCTCCTTCCTCGGTAT-3′	5′-AGCACCGTGTTGGCGTACA-3′

## Data Availability

The original contributions presented in this study are included in the article/Supplementary Material. Further inquiries can be directed to the corresponding author(s).

## Supplementary Materials

The following supporting information can be downloaded at: https://www.mdpi.com/article/10.3390/ani16030515/s1, Table S1: Body weight of fish used in hepatic gene expression analysis expressed as mean ± SD (n = 9 per experimental group). STD, standard diet; HFM, modified diet; 2N, diploid; and 3N, triploid.
