# Supplementary material for: Hepatic Expression of Growth Hormone Receptor (GHrec) and Insulin-like Growth Factor-I (IGF-I) Genes and Cellular Location of IGF-I mRNA in Diploid and Triploid Atlantic Salmon (Salmo salar) Undergoing Parr–Smolt Transformation
Source: Animals (Basel). 2026 Feb 6;16(3):515. doi: 10.3390/ani16030515 (PMC12897259; doi:10.3390/ani16030515)
Supplement: Supplementary file 1 [file animals-16-00515-s001.zip › animals-4065074-supplementary.pdf]

Supplementary Materials

Table S1. Body weight of fish used in hepatic gene expression analysis expressed as mean ± SD (n = 9 per experimental group). STD, standard diet; HFM, modified diet; 2N, diploid; and 3N, triploid.

**Month**	**Diet**	**Ploidy**	**Weight (g)**
October	STD	2N	35.3 ± 13.2
October	HFM	2N	27.5 ± 9.1
October	STD	3N	35.9 ± 13.8
October	HFM	3N	38.2 ± 8.9
November	STD	2N	56.6 ± 8.0
November	HFM	2N	50.6 ± 7.1
November	STD	3N	65.6 ± 11.9
November	HFM	3N	56.1 ± 9.3
December	STD	2N	76.8 ± 10.2
December	HFM	2N	69.2 ± 10.9
December	STD	3N	77.6 ± 11.7
December	HFM	3N	78.3 ± 19.9
